# Identification of a dihydroorotate dehydrogenase inhibitor that inhibits cancer cell growth by proteomic profiling

**DOI:** 10.32604/or.2023.030241

**Published:** 2023-09-15

**Authors:** MAKOTO KAWATANI, HARUMI AONO, SAYOKO HIRANUMA, TAKESHI SHIMIZU, MAKOTO MUROI, TOSHIHIKO NOGAWA, TOMOKAZU OHISHI, SHUN-ICHI OHBA, MANABU KAWADA, KANAMI YAMAZAKI, SHINGO DAN, NAOSHI DOHMAE, HIROYUKI OSADA

**Affiliations:** 1 Biomolecular Characterization Unit, Technology Platform Division, RIKEN Center for Sustainable Resource Science (CSRS), Saitama, 351-0198, Japan; 2 Chemical Resource Development Unit, Technology Platform Division, RIKEN Center for Sustainable Resource Science (CSRS), Saitama, 351-0198, Japan; 3 Chemical Biology Research Group, RIKEN Center for Sustainable Resource Science (CSRS), Saitama, 351-0198, Japan; 4 Molecular Structure Characterization Unit, Technology Platform Division, RIKEN Center for Sustainable Resource Science (CSRS), Saitama, 351-0198, Japan; 5 Institute of Microbial Chemistry (BIKAKEN), Numazu, Microbial Chemistry Research Foundation, Shizuoka, 410-0301, Japan; 6 Division of Molecular Pharmacology, Cancer Chemotherapy Center, Japan Foundation for Cancer Research, Tokyo, 135-8550, Japan; 7 Department of Pharmaceutical Sciences, University of Shizuoka, Shizuoka, 422-8526, Japan

**Keywords:** Anticancer agents, Differentiating agents, Drug target identification

## Abstract

Dihydroorotate dehydrogenase (DHODH) is a central enzyme of the *de novo* pyrimidine biosynthesis pathway and is a promising drug target for the treatment of cancer and autoimmune diseases. This study presents the identification of a potent DHODH inhibitor by proteomic profiling. Cell-based screening revealed that NPD723, which is reduced to H-006 in cells, strongly induces myeloid differentiation and inhibits cell growth in HL-60 cells. H-006 also suppressed the growth of various cancer cells. Proteomic profiling of NPD723-treated cells in ChemProteoBase showed that NPD723 was clustered with DHODH inhibitors. H-006 potently inhibited human DHODH activity *in vitro*, whereas NPD723 was approximately 400 times less active than H-006. H-006-induced cell death was rescued by the addition of the DHODH product orotic acid. Moreover, metabolome analysis revealed that H-006 treatment promotes marked accumulation of the DHODH substrate dihydroorotic acid. These results suggest that NPD723 is reduced in cells to its active metabolite H-006, which then targets DHODH and suppresses cancer cell growth. Thus, H-006-related drugs represent a potentially powerful treatment for cancer and other diseases.

## Introduction

Identification of the molecular targets of bioactive compounds is an important step in chemical genetics and drug discovery processes. Target identification methods are roughly divided into direct and indirect approaches; the former methods are based on direct interactions between proteins and compounds, and the latter on compound-induced cellular phenotypes [1,2]. With the development of omics analysis techniques and technology, a variety of indirect approaches for target identification that use large experimental data sets have been established, including cancer cell, chemical genomic, transcriptomic, proteomic, metabolomic, and cell morphology profiling [3,4].

Pyrimidine and purine nucleotides are crucial for the biosynthesis of DNA, RNA, phospholipids, and glycoproteins. In general, differentiated or resting cells acquire their pyrimidines via the salvage pathway. However, activated immune cells, tumor cells, and many parasites use *de novo* pyrimidine biosynthesis to efficiently produce nucleic acid precursors [5].

Dihydroorotate dehydrogenase (DHODH) is a flavin-dependent enzyme crucial for *de novo* pyrimidine biosynthesis. In the fourth step of the pyrimidine biosynthesis pathway, it catalyzes the conversion of dihydroorotic acid to orotic acid through an oxidative reaction that consumes coenzyme Q/ubiquinone and thereby generates ubiquinol. Human DHODH is a monomeric protein located in the inner mitochondrial membrane and is linked to the respiratory chain via ubiquinone. Recent studies have indicated that DHODH is involved not only in pyrimidine nucleotide biosynthesis but also in various other processes, such as cellular metabolism, growth signaling, ferroptosis, transcription, tumorigenesis, and tumor metastasis [6–13].

Several DHODH inhibitors have been developed against human pathogens [14–17]. However, the clinical application of these inhibitors is limited. Two inhibitors of human DHODH, teriflunomide (A771726) and leflunomide, are used clinically for the treatment of multiple sclerosis and rheumatoid arthritis, respectively [18]. Leflunomide is a prodrug that undergoes non-enzymatic conversion to teriflunomide. It is not always convenient to use teriflunomide or leflunomide in terms of pharmacokinetics and side effects [18].

Modern proteomics techniques can assist in the identification of potentially useful drug compounds. ChemProteoBase is a proteomic profiling system that predicts the molecular targets of bioactive compounds based on proteome analyses using two-dimensional difference gel electrophoresis (2-D DIGE) [19]. The protein expression data that are detected in HeLa cells treated with known inhibitors are classified according to their mechanism of action. Using ChemProteoBase, we identified the mode of action of a variety of small molecules that inhibit cancer cell growth, including glutipyran [20], collismycin A [21], TH287 [22], GN39482 [23], and BNS-22 [24]. The JFCR39 Cell Panel is a cancer cell profiling system that compare the anticancer activities of compounds in various human cancer cells [25]. It was established at the Japanese Foundation for Cancer Research by introducing COMPARE analysis using drug efficacy profiles across the NCI60 panel developed by the National Cancer Institute. Both panels have long been used to predict the mode of action of a compound with unknown functions [26].

Recently, we discovered the novel DHODH inhibitor indoluidin D and its derivatives using JFCR39 Cell Panel and ChemProteoBase [27]. Indoluidin D was originally discovered from a chemical library deposit in the RIKEN Natural Products Depository (NPDepo) [28,29] during cell-based screening for differentiation inducers in human promyelocytic leukemia HL-60 cells. Acute myeloid leukemia (AML) is characterized by arrested differentiation in hematopoietic progenitor cells, and all-*trans* retinoic acid (ATRA) is administered as differentiation-inducing therapy for acute promyelocytic leukemia (APL) [30]. CD11b antigen, a leucocyte differentiation marker, is expressed during myelomonocytic differentiation, and CD14 is upregulated primarily during monocytic differentiation [31,32].

In this study, we report the identification of a novel inhibitor of human DHODH, NPD723, which is more potent than indoluidins. NPD723 was identified by phenotypic screening for differentiation inducers of HL-60 cells. Unlike ATRA, NPD723 strongly not only induced myeloid differentiation but also inhibited the growth of various cancer cells. Phenotypic profiling using ChemProteoBase and the JFCR39 Cell Panel predicted that NPD723 is a DHODH inhibitor. Here, we demonstrate that NPD723 is metabolized in cells to its active form H-006, which then targets DHODH to inhibit cancer cell growth. Our findings will facilitate future investigations into the functions and roles of DHODH in tumors. Our findings suggest that H-006-related drugs may represent a potentially powerful treatment for cancer and other diseases.

## Materials and Methods

### Materials

NPD723 (8-Benzoyl-4-methyl-9-phenyl-2*H*-furo[2,3-*h*]-1-benzopyran-2-one) and H-006 (8-(Hydroxyphenylmethyl)-4-methyl-9-phenyl-2*H*-furo[2,3-*h*]-1-benzopyran-2-one) were obtained from RIKEN NPDepo (RIKEN CSRS, Wako, Japan) [28,29] or synthesized as previously described [33]. ATRA, 1α,25-dihydroxyvitamin D3 [1α,25(OH)_2_D_3_], and A771726 were purchased from Sigma-Aldrich (St. Louis, MO, USA).

### Cell lines

Human cancer cell lines HL-60, K562, Jurkat, HeLa, A549, MCF-7, HepG2, A431, and MIA PaCa-2, along with human normal cell lines WI-38 and 1C3D3, were obtained from the RIKEN Cell Bank (RIKEN BRC, Tsukuba, Japan). Human normal cell line YS-1 was obtained from the JCRB Cell Bank (Osaka, Japan). Human cancer cell lines U937, PC-3, DLD-1, Hep3B, WM266-4, SK-MEL-28, HT-1080, and BxPC-3 were purchased from the American Type Culture Collection (ATCC; Manassas, VA, USA). HL-60, K562, Jurkat, U937, MCF-7, DLD-1, and BxPC-3 cells were cultured in RPMI 1640 (Gibco, Thermo Fisher Scientific, Waltham, MA, USA) containing 10% fetal calf serum (Sigma-Aldrich), 50 units/mL penicillin G (Gibco), and 50 μg/mL streptomycin (Gibco). HeLa, A549, PC-3, HepG2, Hep3B, WM266-4, SK-MEL-28, HT-1080, A431, MIA PaCa-2, and WI-38 cells were cultured in Dulbecco’s modified Eagle’s medium (DMEM; Gibco) containing 10% fetal calf serum, 50 units/mL penicillin G, and 50 μg/mL streptomycin. YS-1 cells were cultured in DMEM containing 5% fetal calf serum, 50 units/mL penicillin G, and 50 μg/mL streptomycin. 1C3D3 cells were cultured in DMEM containing 5% fetal calf serum, 10% newborn bovine serum (SAFC Biosciences, Lenexa, KS, USA), 2.5% horse serum (Gibco), 50 units/mL penicillin G, and 50 μg/mL streptomycin. All cell lines were incubated at 37°C in a humidified atmosphere containing 5% CO_2_.

### Cell differentiation assay

HL-60 cells were treated with test compounds for 96 h. Cells were mixed with a half volume of phosphate-buffered saline (PBS) containing 0.2 mg/mL nitroblue tetrazolium (NBT; Sigma-Aldrich) and 300 nM 12-O-tetradecanoylphorbol-13-acetate (TPA; Sigma-Aldrich) for 1 h at 37°C. Then, NBT-positive blue cells and NBT-negative cells were counted microscopically using a hemocytometer. NBT reduction activity was calculated using the following formula: (NBT-positive cells)/[(NBT-positive cells) + (NBT-negative cells)] × 100.

### Cell proliferation assay

The cell proliferation assay was performed using Cell Count Reagent SF (Nacalai Tesque, Kyoto, Japan) as previously described [27]. Briefly, the cells were exposed to NPD723, H-006, or A771726 for 72 h. After adding WST-8 and incubating at 37°C for 1 h, cell proliferation was measured using a microplate reader (Varioskan LUX, Thermo Fisher Scientific) based on absorbance at 450 nm.

### Cell death assay

Cell death was assessed using a trypan blue dye exclusion test. HL-60 cells were treated with 100 nM H-006 or 250 μM A771726 in the presence or absence of the indicated concentrations of orotic acid or dihydroorotic acid for 48 h. Cells were then stained with a trypan blue solution (Sigma-Aldrich) and counted. Cell death (%) was determined as the proportion of trypan blue-stained cells among all cells.

### Analysis of cell surface antigens

HL-60 cells were seeded in a 12-well plate and cultured for 96 h with NPD723. Then, cells were suspended in PBS containing 1% bovine serum albumin and incubated with 1 μg anti-CD11b (Beckman Coulter, Brea, CA, USA) and anti-CD14 (Beckman Coulter) antibodies for 3 h on ice. After incubation, cells were washed with PBS once and incubated with secondary antibodies (FITC-conjugated anti-mouse IgG_1_ and phycoerythrin [PE]-conjugated anti-mouse IgG_2a_; Beckman Coulter). Cells were washed with PBS and then fixed in PBS containing 2% paraformaldehyde. Fluorescence was detected using a Cytomics FC 500 flow cytometer (Beckman Coulter).

### Cell cycle analysis

HL-60 cells were treated with NPD723 for 48 h. After washing with PBS and fixing in 70% ethanol, cells were washed twice with PBS and incubated in PBS containing 50 μg/mL propidium iodide and 2 μg/mL RNase A (Nacalai Tesque) for 30 min. The DNA content of the cells was determined using a Cytomics FC500 flow cytometer (Beckman Coulter).

### Semi-quantitative RT-PCR

HL-60 cells were treated with 3 nM NPD723, 1 μM ATRA, or 1 μM 1α,25(OH)_2_D_3_ for the indicated times (Suppl. Fig. S1). Total RNA from the cells was isolated using Isogen (Nippon Gene Co., Ltd., Tokyo, Japan). The first-strand cDNA was synthesized with SuperScript II reverse transcriptase (Invitrogen, Carlsbad, CA, USA) using oligo-dT primer. The sequences of the primers were as follows: retinoic acid receptor β (RARβ), forward (5′-GGAGACCGCCAGGACCTTGAGG-3′), reverse (5′-GGACTGTGCTCTGCTGTGTTCCC-3′); 1α,25(OH)_2_D_3_ 24-hydroxylase (24OHase), forward (5′-CCTGGAAGGGGAAGACTGGC-3′), reverse (5′-GTGTCCCTGCCAGACCTTGG-3′); c-myc, forward (5′-CTCCTGGCAAAAGGTCAGAG-3′), reverse (5′-AGCTTTTGCTCCTCTGCTTG-3′); β-actin, forward (5′-CAAGAGAGGCATCCTCATCC-3′), reverse (5′-CGTACATGGCTGGGGTGTTG-3′).

### Metabolite analysis of NPD723

HL-60 cells (1 × 10^6^ cells) were treated with 10 μM NPD723 for 1 or 8 h, or with DMSO for 8 h as a control. Cells were washed with PBS once and added to a mixture of chloroform and water (1:1, v/v). After vortexing, samples were centrifuged at 2,400 × *g* for 3 min to obtain chloroform extracts. The chloroform extracts were then dried, dissolved in methanol, and analyzed via liquid chromatography-mass spectrometry (LC/MS) using a Waters UPLC H-class system (Waters, Milford, MA, USA) fitted to a mass spectrometer (API3200; Sciex, Framingham, MA, USA). The following conditions were used: column, Waters UPLC BEH C18 (φ2.1 × 50 mm, 1.7 μm); solvent A, 0.05% formic acid in water; solvent B, acetonitrile; gradient, 5–100%B/0–4 min, 100–100%B/4–6 min; flow rate, 0.5 mL/min; scan mode, Q1 (positive mode).

### ChemProteoBase analysis

ChemProteoBase analysis of NPD723-treated cells was performed as previously described [34]. Briefly, HeLa cells were treated with 100 μM NPD723 for 18 h. Then, the cell lysate was subjected to proteome analysis by 2-D DIGE. Among over 1,000 spots detected in each 2-D gel, 296 common spots were used to perform hierarchical clustering analysis.

### The JFCR39 cell panel

Analysis using the JFCR39 Cell Panel was performed as previously described [25,35]. Briefly, the JFCR39 cancer cell lines were treated with NPD723 or H-006 for 48 h. Cell growth was analyzed via sulforhodamine B assay. A COMPARE analysis was performed by calculating Pearson’s correlation coefficients (*r*) between the 50% growth inhibition (GI_50_) mean graphs of the compounds. Pearson’s correlation coefficients were used determine the degree of similarity.

### DHODH enzyme assay

An DHODH enzyme assay was performed using dichloroindophenol (DCIP) [36,37] as previously described [27]. Recombinant human *N*-terminal His-tagged DHODH with a deletion in the transmembrane domain (DHODH/ΔTM; Met29–Arg395) was preincubated with NPD723 or H-006 in a buffer containing 50 mM Tris-HCl (pH 8.0), 150 mM KCl, 100 μM coenzyme Q10 (Sigma-Aldrich), 0.05% Triton X-100, and 200 μM DCIP (Sigma-Aldrich) at 25°C for 30 min. Then, 500 μM dihydroorotic acid (Sigma-Aldrich) were added to the sample, and absorbance was measured at 650 nm with a microplate reader (Varioskan LUX, Thermo Fisher Scientific) at 25°C for 10 min.

### Metabolome analysis of H-006-treated cells

A549 cells (5 × 10^6^ cells) were treated with 100 nM H-006 for 24 h. Then, cells were washed with 5% mannitol solution twice, treated with 1.3 mL methanol, and collected with a cell scraper. The cell extracts were subjected to capillary electrophoresis-time-of-flight mass spectrometry (CE-TOFMS). Metabolome analysis was performed by Human Metabolome Technologies, Inc. (Yamagata, Japan) as previously described [38].

### Animal experiments

Animal experiments were performed as previously described [27]. Seven-week-old female BALB/c nude mice (Charles River Laboratories Japan, Inc., Yokohama, Japan) were used for the experiments. PC-3 cells (0.3 mL of 2.66 × 10^7^ cells/mL) were mixed with 0.5 mL Matrigel (growth factor-reduced; BD Biosciences, Franklin Lakes, NJ, USA). A 100 μL suspension containing 10^6^ PC-3 cells was subcutaneously injected into the left lateral flank of each mouse. The mice were divided into vehicle control, H-006-treated (25 mg/kg), and cisplatin (CDDP, Nippon Kayaku Co., Ltd., Tokyo, Japan) (5 mg/kg) groups. H-006 was intraperitoneally injected once daily on days 7–14, 17–21, 24–28, and 31–34. CDDP was intravenously injected once daily on days 7, 14, 21, and 28. The mice were weighed and tumor growth was monitored and recorded via caliper measurements. The mice were sacrificed on day 35 after cell implantation, and their tumors were removed and weighed.

### Statistical analysis

Data were expressed as mean ± SD of at least three independent experiments. Statistical analysis was performed by using ANOVA followed by the Tukey-Kramer test (for cell death assay), Welch’s *t*-test (for metabolome analysis), or Dunnett’s test (for animal experiments). A value of *p* < 0.05 was considered statistically significant.

## Results

### NPD723 potently induces myeloid differentiation

**Figure 1:**
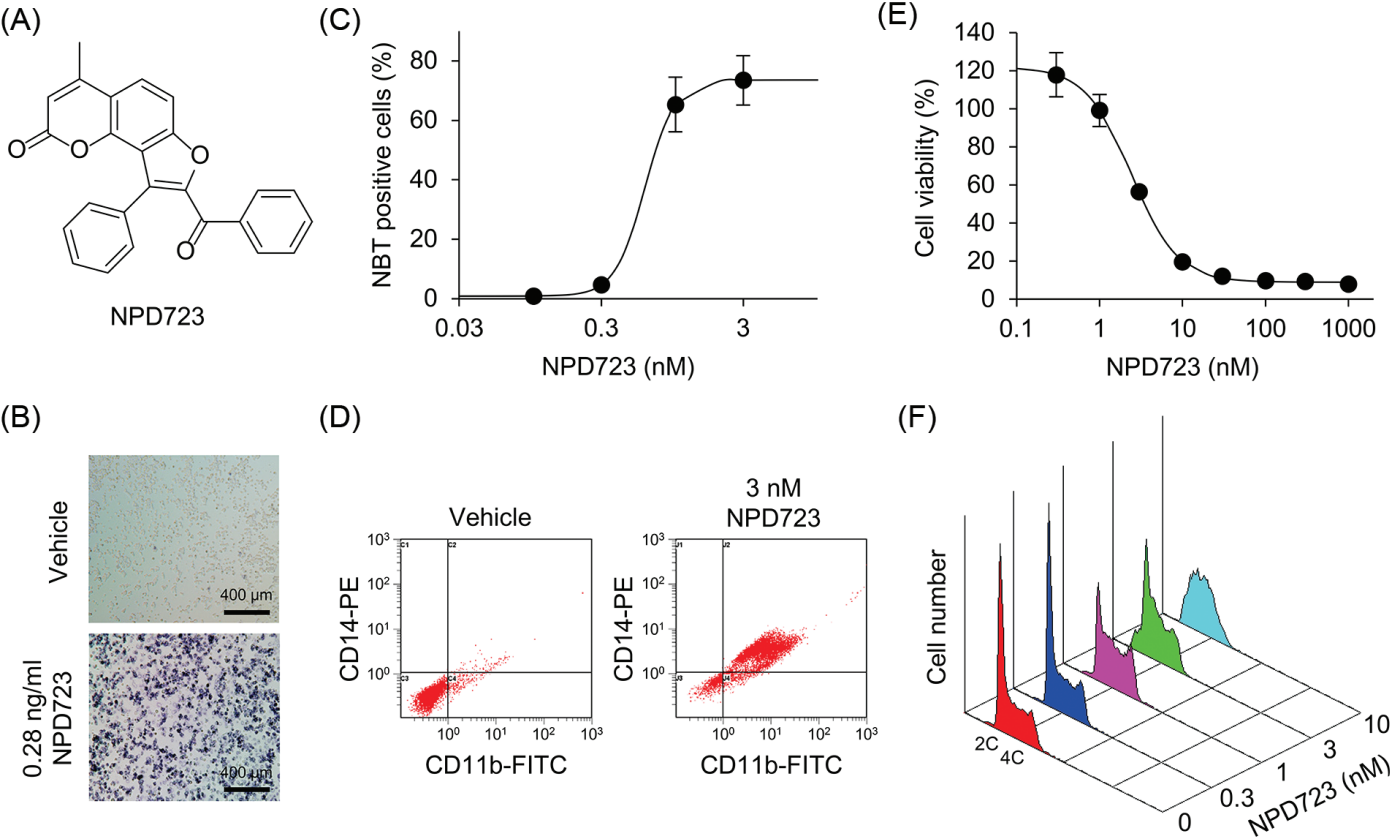
NPD723 induces myeloid differentiation. (A) Structure of NPD723. (B) NBT-positive HL-60 cells induced by NPD723. Scale bar indicates 400 μm. (C) Cell differentiation of HL-60 cells after NPD723 treatment was assessed by NBT assay. (D) NPD723-induced immunophenotypic changes were analyzed by flow cytometry. (E) Cell growth of HL-60 cells after NPD723 treatment was analyzed by WST-8 assay. (F) Cell cycle of HL-60 cells after NPD723 treatment was analyzed by flow cytometry.

To discover novel myeloid differentiation inducers, we performed cell-based screening by measuring the functional maturation of HL-60 cells in an NBT reduction assay. We screened 6,656 compounds in the chemical library of RIKEN NPDepo and obtained NPD723 as a hit (Figs. 1A and 1B). NPD723 strongly induced cell differentiation, with a half-maximal effective concentration (EC_50_) value of 0.83 nM (Fig. 1C). The NBT reduction activity of NPD723 was equal to or greater than that of ATRA (EC_50_, 1.6 nM) and 1α,25(OH)_2_D_3_ (EC_50_, 182 nM) [27]. Flow cytometric analysis showed that NPD723 treatment increased the expression of both CD11b and CD14 in HL-60 cells, suggesting that NPD723 induced monocytic differentiation (Fig. 1D). NPD723, unlike ATRA and 1α,25(OH)_2_D_3_, induced neither RARβ nor 24OHase expression, whereas all three compounds similarly downregulated c-myc levels, as measured by semi-quantitative RT-PCR (Suppl. Fig. S1). This result indicated that NPD723 had a different mechanism of action from that of ATRA and 1α,25(OH)_2_D_3_.

We also found that NPD723 exhibited potent anti-proliferative activity. NPD723 inhibited the growth of HL-60 cells with an IC_50_ value of 3.7 nM (Fig. 1E). When HL-60 cells were treated with NPD723 for 48 h, flow cytometric analysis showed that the relative proportion of cells in the S phase increased compared with that in control samples (Fig. 1F). Thus, NPD723 potently induced myeloid differentiation and inhibited cell growth in HL-60 cells.

### NPD723 is reduced to H-006

**Figure 2:**
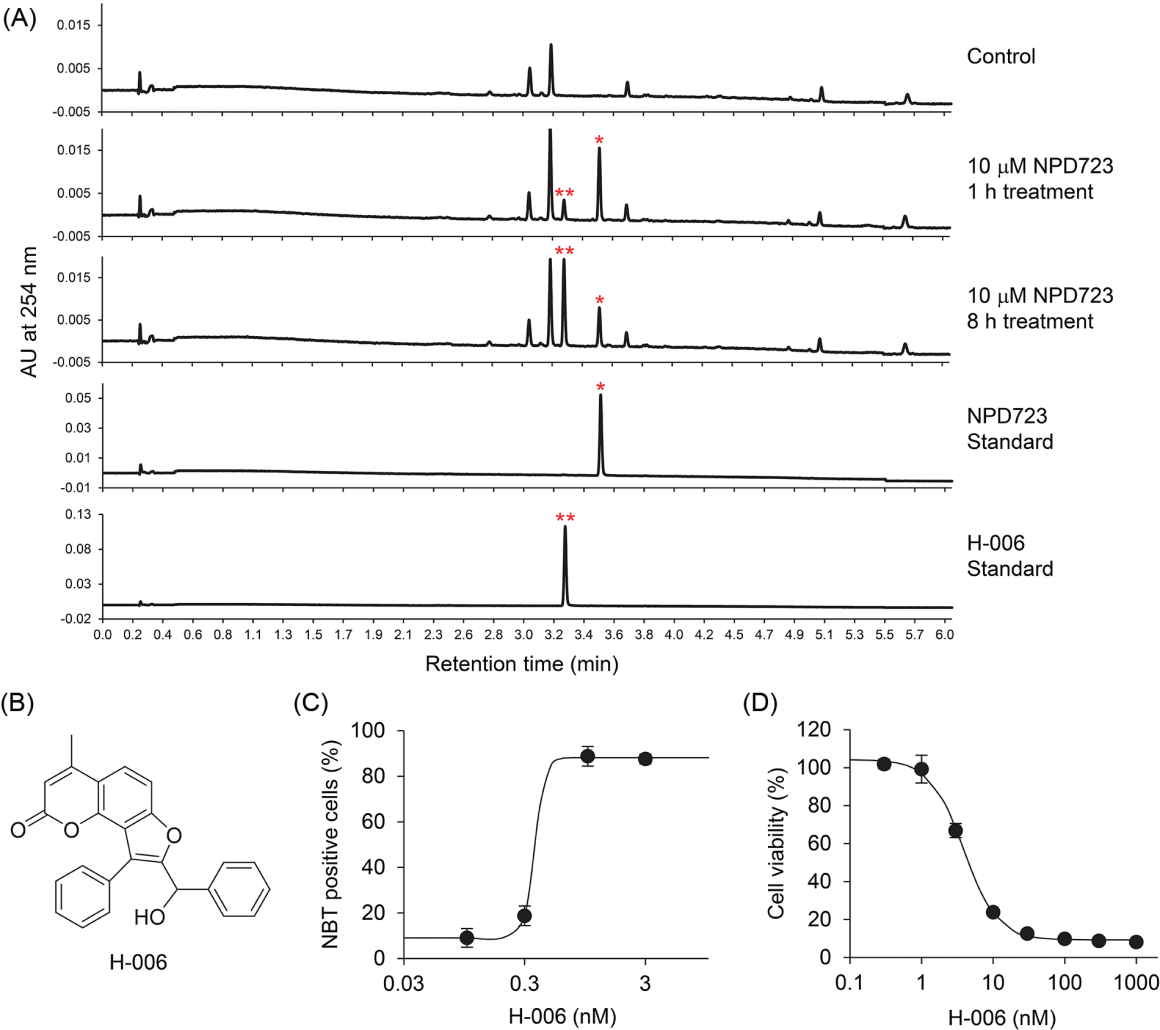
NPD723 is metabolized to H-006. (A) UV chromatogram of NPD723-treated cell extracts. NPD723 standard, 3 nM NPD723 dissolved in DMSO; H-006 standard, 3 nM H-006 dissolved in DMSO; *, peak corresponding to NPD723; **, peak corresponding to H-006. (B) Structure of H-006. (C) Cell differentiation of HL-60 cells after H-006 treatment was assessed by NBT assay. (D) Cell growth of HL-60 cells after H-006 treatment was analyzed by WST-8 assay.

To investigate whether NPD723 is metabolized in cells, we analyzed the extracts of NPD723-treated cells using LC/MS. HL-60 cells were treated with NPD723 for 1 and 8 h, extracted with chloroform, and subjected to LC/MS analysis. UV chromatography showed a peak in retention time at 3.52 min corresponding to NPD723; this shifted to a peak at 3.27 min in a time-dependent manner (Fig. 2A and Suppl. Fig. S2). The shifted peak was identical to the characteristic peak of H-006, a reduced form of NPD723 (Fig. 2B and Suppl. Fig. S2). H-006 induced cell differentiation of HL-60 cells with an EC_50_ value of 0.63 nM (Fig. 2C) and inhibited cell growth with an IC_50_ value of 4.8 nM (Fig. 2D), showing almost equal activity to that of NPD723 in cells.

### H-006 inhibits the growth of various cancer cells

To examine the antiproliferative activity of H-006, we treated various cancer cell lines, including lung, breast, prostate, colon, liver, skin, and pancreas cancer cells, with H-006. H-006 exhibited potent antiproliferative activity against acute T cell leukemia Jurkat, histiocytic lymphoma U937, lung carcinoma A549, prostate adenocarcinoma PC-3, and fibrosarcoma HT-1080 cells (Table 1). However, it was less effective against normal lung fibroblast WI-38, normal stomach fibroblast YS-1, normal pancreatic cell 1C3D3, colorectal adenocarcinoma DLD-1, and pancreatic adenocarcinoma BxPC-3 cells (Table 1).

**Table 1:** H-006 inhibits cell growth of various cancer cells

Cell line	Origin	IC_50_ (nM)
WI-38	Normal lung fibroblast	>10,000
YS-1	Normal stomach fibroblast	>10,000
1C3D3	Normal pancreatic cell	>10,000
K562	Chronic myeloid leukemia	3.0 ± 0.3
Jurkat	Acute T cell leukemia	0.23 ± 0.11
U937	Histiocytic lymphoma	0.50 ± 0.01
HeLa	Cervical adenocarcinoma	5.2 ± 0.1
A549	Lung carcinoma	2.1 ± 0.2
MCF-7	Breast adenocarcinoma	4.7 ± 0.3
PC-3	Prostate adenocarcinoma	1.7 ± 0.2
DLD-1	Colorectal adenocarcinoma	4,577 ± 785
HepG2	Hepatocellular carcinoma	24.6 ± 1.1
Hep3B	Hepatocellular carcinoma	188 ± 115
WM266-4	Melanoma	3.7 ± 0.1
SK-MEL-28	Melanoma	11.5 ± 5.0
HT-1080	Fibrosarcoma	0.45 ± 0.02
A431	Epidermoid carcinoma	3.3 ± 0.4
MIA PaCa-2	Pancreatic carcinoma	79.4 ± 40.6
BxPC-3	Pancreatic adenocarcinoma	5,955 ± 191

### ChemProteoBase prediction: NPD723 targets DHODH

**Figure 3:**
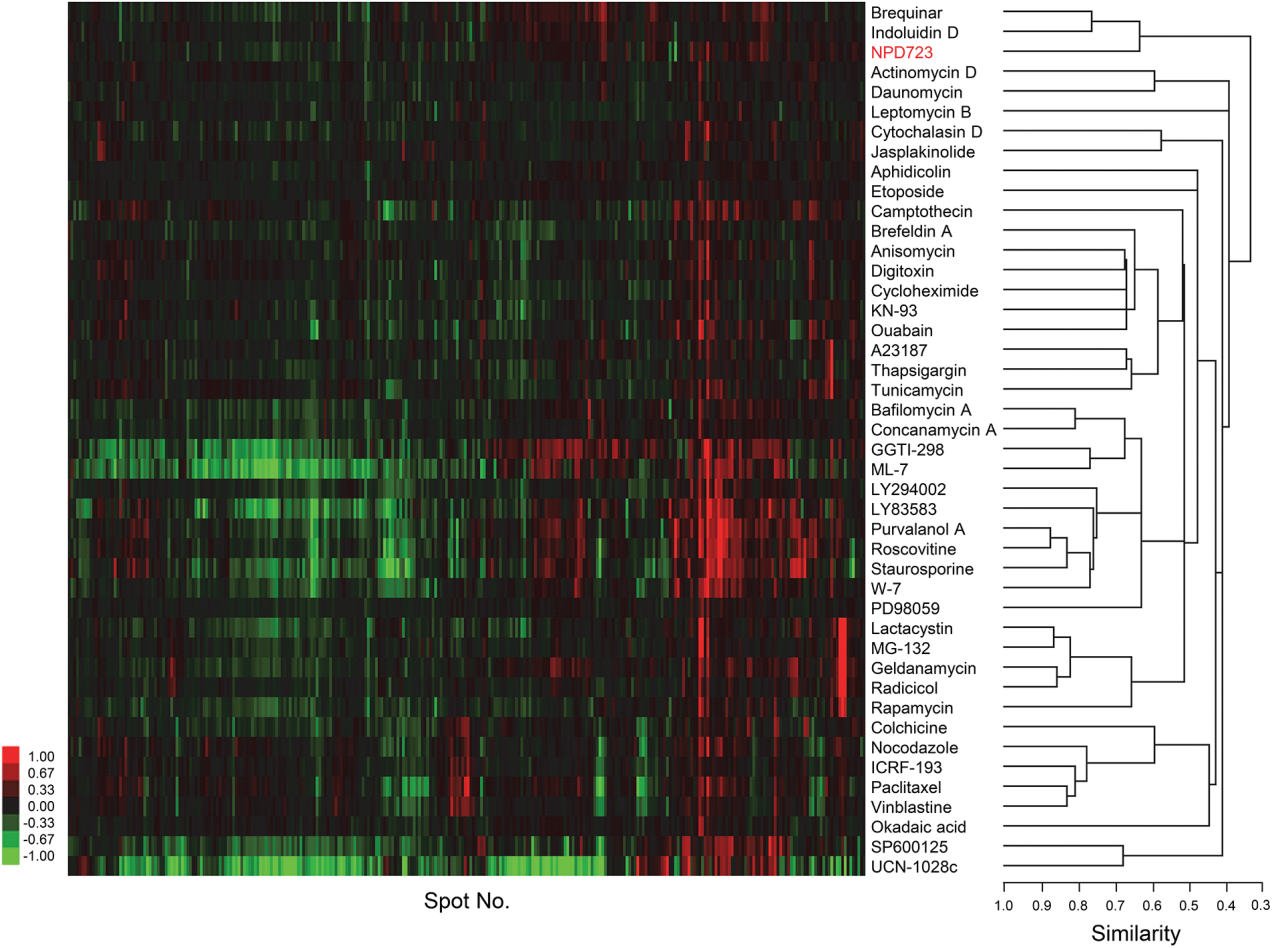
ChemProteoBase predicts NPD723 as a DHODH inhibitor. Hierarchical clustering of NPD723-treated HeLa cells by ChemProteoBase. In the heat map, log-fold (natural base) of normalized volume is shown on a colored scale. The x-axis represents quantitative data for 296 common spots derived from NPD723 and those for 43 well-characterized compounds.

To predict the targets of NPD723, we performed proteomic profiling on NPD723-treated cells via ChemProteoBase. HeLa cells were treated with NPD723 for 18 h, and the lysates were subjected to 2-D DIGE. Using 296 common spots matching in all gel images, we performed a hierarchical cluster analysis of 43 standard inhibitors in the database against NPD723. NPD723 clustered with DHODH inhibitors brequinar and indoluidin D (Fig. 3).

We then used the JFCR39 Cell Panel to conduct sensitivity profiling on cells treated with NPD723 and H-006 for 48 h. Cell viability was assessed by sulforhodamine B assay. The antiproliferative profiles of NPD723 and H-006 across the JFCR39 cell lines were similar to those of indoluidin D and brequinar (Suppl. Fig. S3). The Pearson’s correlation coefficients (*r*) of H-006, indoluidin D, and brequnar against NPD723 were 0.792, 0.735, and 0.648, respectively (Suppl. Fig. S3). Collectively, these results suggested that NPD723 targeted DHODH.

### H-006 is a DHODH inhibitor

**Figure 4:**
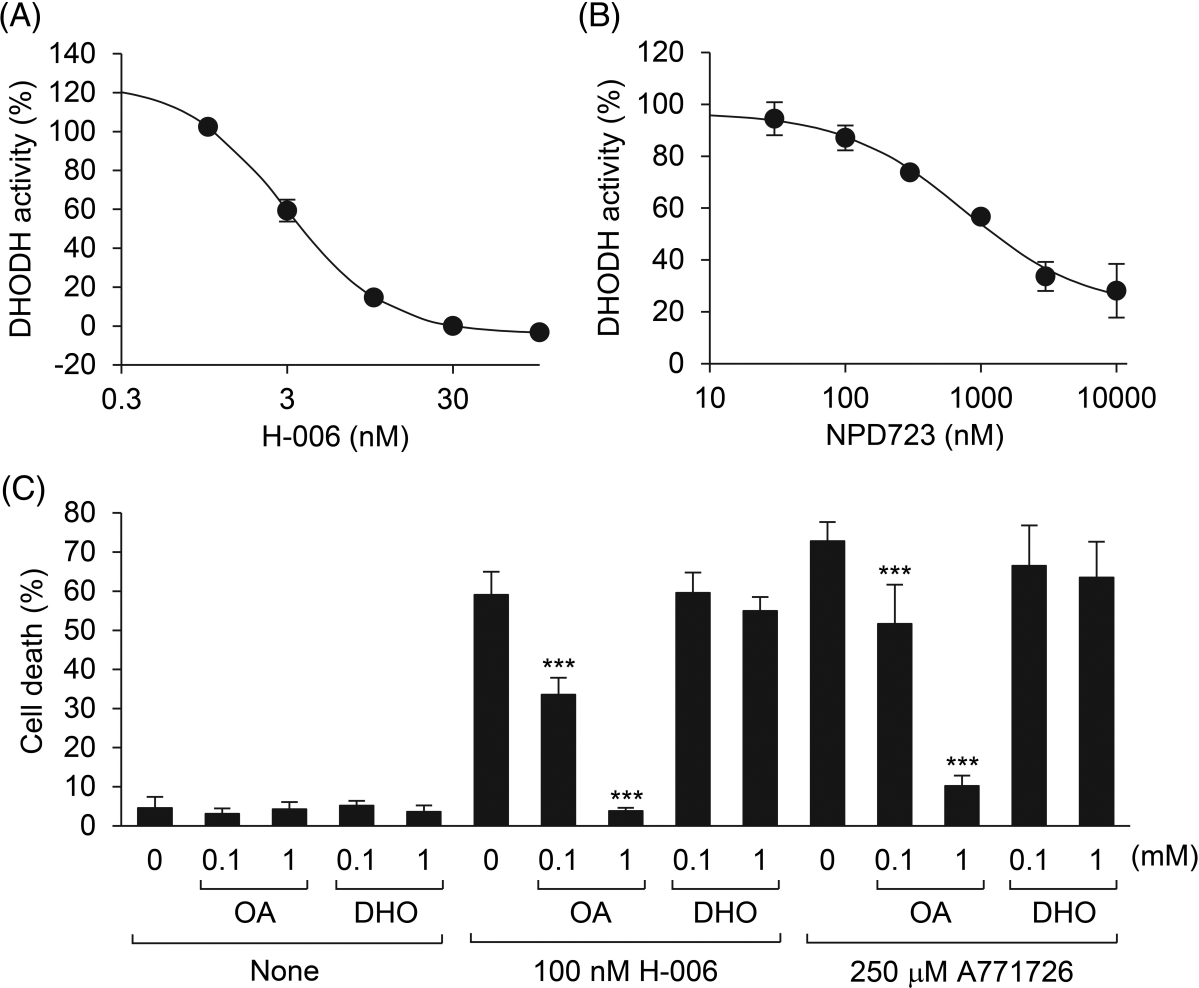
H-006 is a potent and selective DHODH inhibitor. (A, B) Effect of H-006 (A) and NPD723 (B) on DHODH enzyme activity. (C) Cell death of HL-60 cells after the treatment of H-006 or A771726 in the presence of orotic acid or dihydroorotic acid was analyzed by a trypan blue dye exclusion assay. ***, *p* < 0.001. OA, orotic acid; DHO, dihydroorotic acid.

To test whether NPD723 and H-006 inhibit DHODH activity, we performed a DHODH enzyme assay using recombinant human His-tagged DHODH/ΔTM. H-006 inhibited DHODH in a concentration-dependent manner, with an IC_50_ value of 3.8 nM (Fig. 4A). On the contrary, NPD723 showed weak inhibitory activity, with an IC_50_ value of 1,523 nM; NPD723 was therefore approximately 400 times less active than H-006 (Fig. 4B). These results, together with those presented in Fig. 2, suggested that NPD723 was metabolized to H-006, which acted as the active form of NPD723.

To examine the specificity of H-006 in cells, we conducted rescue experiments with the DHODH product orotic acid. H-006-induced cell death in HL-60 cells was rescued by the addition of orotic acid in a concentration-dependent manner (Fig. 4C). However, cell death was not rescued by the addition of the DHODH substrate dihydroorotic acid (Fig. 4C). Similar results were obtained when HL-60 cells were treated with 250 μM A771726 (Fig. 4C and Suppl. Fig. S4). The addition of orotic acid also suppressed H-006-induced cell differentiation (Suppl. Fig. S5). Thus, H-006 was a potent and selective DHODH inhibitor.

### H-006 promotes cellular dihydroorotic acid accumulation

**Figure 5:**
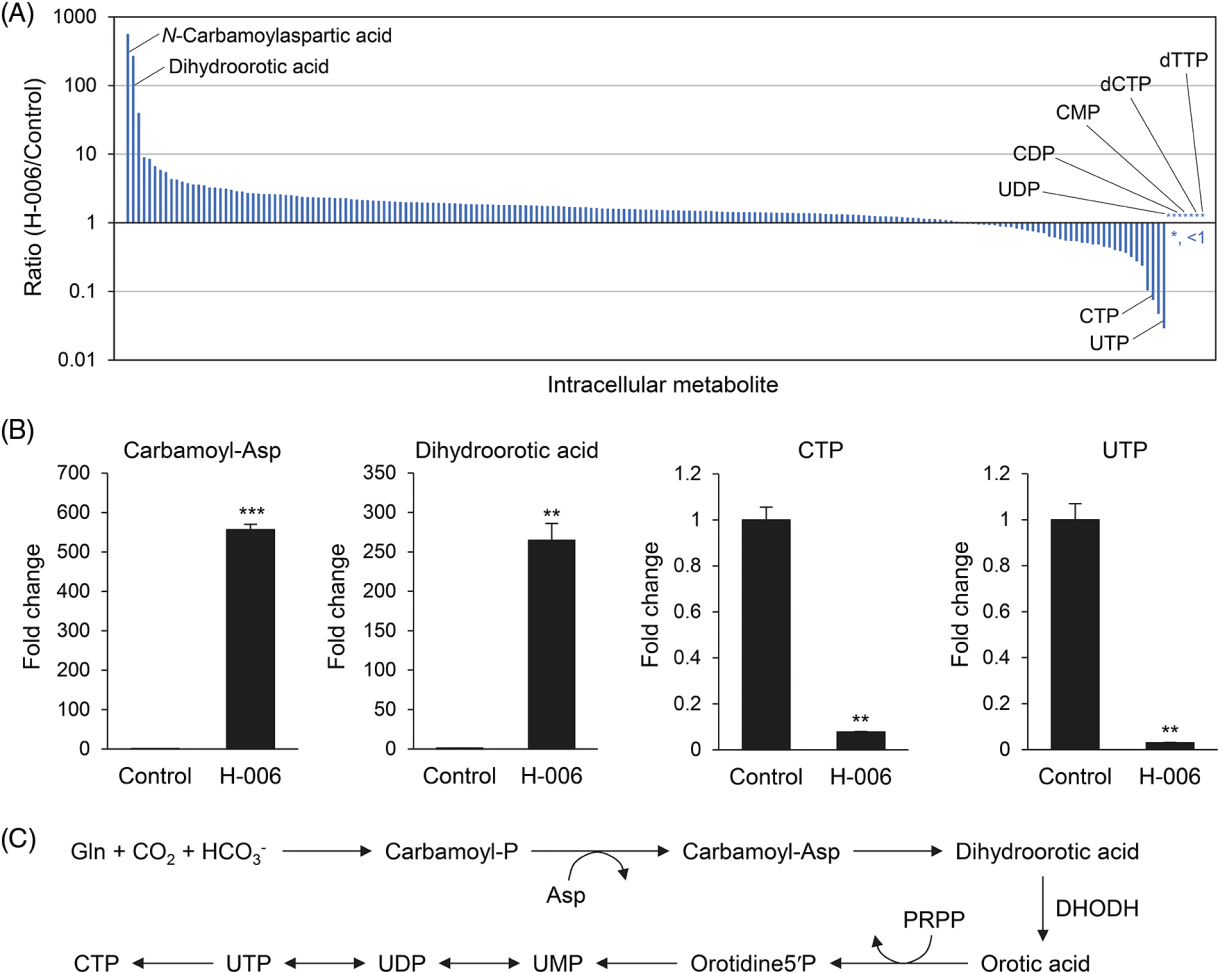
H-006 treatment causes dihydroorotic acid accumulation in A549 cells. (A) Ratios of intracellular metabolites (H-006/Control) were used to construct a waterfall plot. A total of 201 metabolites were arranged in decreasing order of ratio for each metabolite. *, <1. See also Suppl. Table S1. (B) Changes in *N*-carbamoylaspartic acid, dihydroorotic acid, cytidine triphosphate, and uridine triphosphate contents upon H-006 treatment in A549 cells. **, *p* < 0.01; ***, *p* < 0.001. Carbamoyl-Asp, *N*-carbamoylaspartic acid. (C) *De novo* pyrimidine biosynthesis pathway. PRPP, phosphoribosyl pyrophosphate; Orotidine5′P, orotidine-5-monophosphate; UMP, uridine-5′-monophosphate.

To examine the effects of H-006 on cell metabolism, we conducted a metabolome analysis of H-006-treated A549 cells. In total, 201 metabolites were detected with absolute quantitative values (Suppl. Table S1). H-006 administration was associated with a marked accumulation of dihydroorotic acid (265-fold) and its upper metabolite *N*-carbamoylaspartic acid (556-fold) compared to that of the control (Figs. 5A and 5B). In addition, H-006 administration resulted in a marked decrease in the levels of many pyrimidine nucleotides, including those of uridine triphosphate and cytidine triphosphate (Figs. 5A and 5B). Several pyrimidine nucleotides, such as uridine diphosphate, cytidine diphosphate, and cytidine monophosphate, were not detected in H-006-treated cells (Fig. 5A and Suppl. Table S1). These results indicated that H-006 specifically affected the *de novo* pyrimidine biosynthetic pathway (Fig. 5C).

**Figure 6:**
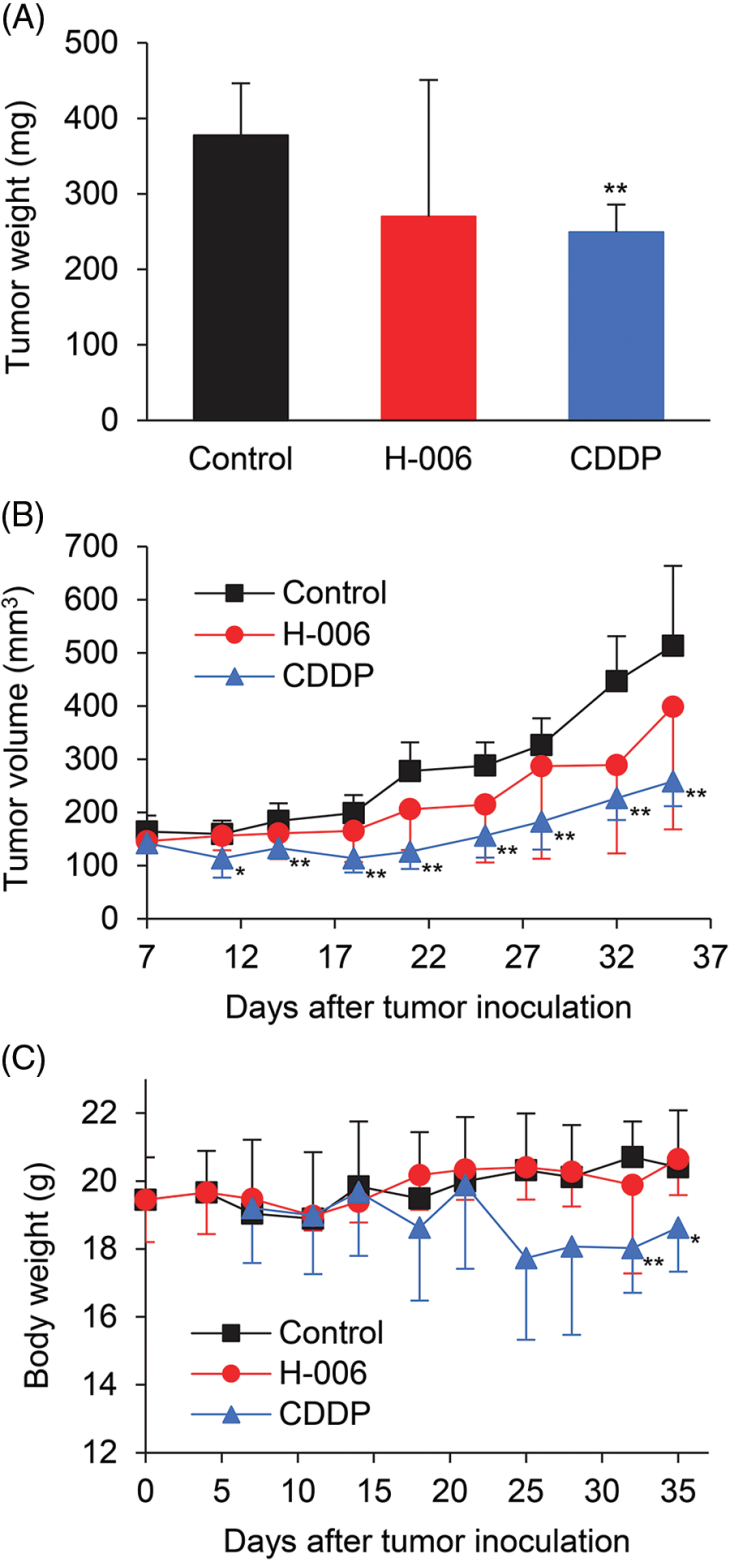
Effect of H-006 on tumor growth in the xenograft model of prostate cancer cells. (A) Tumor weight of PC-3 xenografts on day 35. (B) Tumor volume of PC-3 xenografts. (C) Body weight change in mice. *, *p* < 0.05; **, *p* < 0.01 *vs*. control.

We next evaluated the anti-tumor efficacy of H-006 using an PC-3 xenograft mouse model. Because H-006 strongly inhibited cell growth of PC-3 cells (Table 1), we selected the PC-3 xenograft model. The mice were treated intraperitoneally with 25 mg/kg H-006 or intravenously with 5 mg/kg cisplatin (CDDP) as a positive control group. Although no significant differences were observed between control and H-006-treated groups, H-006 administration was associated with inhibited growth of PC-3 xenografts, as judged by tumor weight and volume (Figs. 6A and 6B). H-006 treatment did not cause significant body weight loss, while CDDP did in the late period of treatment (Fig. 6C).

## Discussion

We have shown that NPD723 is reduced to H-006, which targets DHODH, resulting in the induction of myeloid differentiation in HL-60 cells and the inhibition of growth in various cancer cell lines. NPD723 and H-006 had similar activities in myeloid differentiation and cell growth inhibition (Figs. 1 and 2). However, NPD723 exhibited very weak inhibitory activity against DHODH *in vitro* (Fig. 4B), indicating that NPD723 itself is not active in cells. Our findings show that H-006 acts as a potent and selective DHODH inhibitor in cells. This conclusion is supported by the following findings: (i) H-006 inhibited DHODH *in vitro* and suppressed cell growth in cells at nanomolar ranges of concentration (Fig. 4A and Table 1); (ii) H-006 led to specific, high accumulation of the DHODH substrate dihydroorotic acid in cells (Fig. 5); and (iii) the effects of H-006 were significantly attenuated by the addition of the DHODH product orotic acid to cells (Fig. 4C and Suppl. Fig. S5). Based on our experiments, the inhibitory activity of H-006 against DHODH is about 100-fold stronger than that of A771726, which exhibited an IC_50_ value of 411 nM [27] (Fig. 4).

H-006 was not effective against DLD-1 and BxPC-3 cells (Table 1). DHODH is expressed in every cell and every tissue. Although the detailed mechanism is unknown, DLD-1 and BxPC-3 cells may not be dependent on *de novo* pyrimidine biosynthesis for their growth.

Metabolome analysis showed that *N*-acetylneuraminic acid levels increased 39-fold in H-006-treated cells relative to levels in control cells (Fig. 5 and Suppl. Table S1). *N*-acetylneuraminic acid is the most abundant sialic acid and is involved in nucleotide sugar metabolism [39]. The accumulation of *N*-acetylneuraminic acid may have occurred due to the perturbation of sugar metabolism by the deficiency of pyrimidine nucleotides caused by DHODH inhibition. Indeed, the levels of other sugar metabolites, including *N*-acetylglucosamine 6-phosphate (increase, *p* < 0.01), *N*-acetylglucosamine 1-phosphate (increase, *p* < 0.001), and uridine diphosphate-*N*-acetylglucosamine (decrease, *p* < 0.001), were also altered significantly by H-006 treatment (Suppl. Table S1).

NPD723/H-006, as well as ML390 [9], isobavachalcone [40], BAY 2402234 [41], and indoluidins [27], induce myeloid cell differentiation (Figs. 1 and 2). Thus, DHODH inhibitors similarly induce myeloid differentiation, but the detailed mechanism(s) involved remain unknown. DHODH inhibitors perturb the biosynthesis of DNA, RNA, glycoproteins, and phospholipids by depleting pyrimidine nucleotide pools [12,15]. Moreover, co-expression network analysis has indicated that various genes are associated with DHODH [12,15]. Therefore, the mechanism underlying compound-induced myeloid differentiation is likely very complex. As H-006-induced cell differentiation was rescued by orotic acid in the present study, it is likely that differentiation is controlled downstream of this pathway. Like ATRA and 1α,25(OH)_2_D_3_, NPD723 and indoluidin E have been shown to downregulate the expression of the transcription factor c-Myc [27] (Suppl. Fig. S1). c-Myc is involved in myeloid leukemogenesis, and a small-molecule c-Myc inhibitor induces myeloid differentiation [42]. Therefore, the downregulation of c-Myc may be involved in DHODH inhibitor-induced myeloid differentiation observed here.

Recent studies have indicated that a DHODH blockade can be effective for the treatment of COVID-19 [43–45]. DHODH inhibitors suppress the replication of SARS-CoV-2 by reducing the amount of pyrimidine nucleotide pools in the host cells. DHODH inhibitors also can block cytokine release by affecting immune cells. Therefore, it would be reasonable to test the biological activity of H-006 against viral infection with SARS-CoV-2. It has been reported that NPD723 (8-Benzoyl-4-methyl-9-phenyl-2*H*-furo[2,3-*h*]-1-benzopyran-2-one) and H-006 (8-(Hydroxyphenylmethyl)-4-methyl-9-phenyl-2*H*-furo[2,3-*h*]-1-benzopyran-2-one) exhibit anti-viral activity against influenza viruses [33,46]. This may be due to the inhibition of host DHODH.

This study demonstrates the usefulness of ChemProteoBase and JFCR39 Cell Panel profiling systems for drug target identification. When we obtained NPD723 as a hit via cell-based screening, we first tried to identify the target proteins by affinity purification using photo-crosslinked NPD723 beads [47]. However, we were not able to identify DHODH as the binding protein. This may be due to the following reasons: first, NPD723 is a prodrug of H-006 and a very weak inhibitor of DHODH. Additionally, DHODH is a membrane-associated protein, which may be difficult to identify using an affinity-based direct approach. Hence, multiple approaches may be necessary to successfully identify the target molecules of bioactive small molecules.

In the present study, H-006 tended to suppress tumor growth but did not show significant anti-tumor activity *in vivo* (Fig. 6). We performed a pharmacokinetic analysis of H-006 in Balb/c mice. The t_1/2_ in plasma after a single intravenous administration of H-006 at 3 mg/kg was 12 min. We also performed a metabolic stability test of H-006 in mouse liver microsomes. The half-life of H-006 in mouse liver microsomes was 4.8 min. These results suggest that H-006 did not show significant anti-tumor activity due to its low bioavailability, a result of its poor metabolic stability. Hence, structural optimization of H-006 is needed for future drug development. The development of a crystal DHODH structure complexed with H-006 may represent a method to synthesize H-006 derivatives. From the viewpoint of cancer therapy, the investigation of H-006 complexes with clinically used anticancer drugs may represent an effective drug development method. Indeed, DHODH inhibitors such as brequinar and leflunomide exhibit synergistic effects with 5-fluorouracil [48], gemcitabine [49], or doxorubicin [50] on the growth inhibition of cancer cells. As several oncogenic backgrounds share synthetic lethality with DHODH [15], it will be important to select tumor types that are highly sensitive to DHODH inhibitors and to combine molecularly targeted drugs with DHODH inhibitors.

In summary, we demonstrated that NPD723 is metabolized to H-006, which acts as a potent and selective inhibitor of DHODH, thereby suppressing cancer cell growth and inducing myeloid differentiation. Moreover, our study has shown that ChemProteoBase and the JFCR39 Cell Panel are useful tools for the prediction of cellular targets of bioactive compounds. Although the detailed mechanism of action of H-006 remains to be elucidated, our findings will facilitate the study of tumor metabolism and differentiation and support the development of therapeutic agents.

## Supplementary Materials

FIGURE S1 Effect of NPD723 on gene expression in HL-60 cells. HL-60 cells were treated with 3 nM NPD723, 1 μM ATRA, or 1μM 1α,25(OH)_2_D3 for the indicated times. The gene expression levels were determined by semi-quantitative RT-PCR.

FIGURE S2 UV and MS spectra of NPD723 and H-006. (A–C) Supplemental data of Fig. 2A. (A) NPD723 standard. (B) H-006 standard. (C) Cell extracts treated with 10 μM NPD723 for 8 h.

FIGURE S3 JFCR39 panel predicts that NPD723 and H-006 target DHODH. Fingerprint shows the differential growth inhibition pattern of compounds tested against JFCR39 cell lines. X-axis represents the difference in logarithmic scale between mean LogGI50 for 39 cell lines and LogGI50 for each cell line. Bars to the right of 0 indicate cell lines sensitive to the compound. Bars to the left of 0 indicate resistance. MG-MID, mean LogGI50 for 39 cell lines; Delta, difference between MG-MID and LogGI50 for most sensitive cell line; Range, difference in LogGI50 between most resistant and most sensitive cell lines. Br, breast; CNS, central nervous system; Co, colorectal; Lu, lung; Me, melanoma; Ov, ovarian; Re, renal; St, stomach; xPg, prostate.

FIGURE S4 A771726 inhibits cell growth in cancer cells. (A) Structure of leflunomide and A771726. (B–D) HL-60cells (B), A549cells (C), and Jurkat cells (D) were treated with A771726 for 72 h, and cell growth was analyzed using WST-8 assay. Data are means ± SD (n = 3).

FIGURE S5 Orotic acid rescues cells from H-006-induced cell differentiation. HL-60 cells were treated with 1 mM orotic acid for 1 h and then treated with the indicated concentrations of H-006 for 96 h. After NBT staining, cells were enumerated. Data are means ± SD (n = 3). Statistical analysis was performed by using ANOVA followed by the Tukey-Kramer test. *, *p* < 0.05; ***, *p* < 0.001 *vs*. control(-OA). OA, orotic acid.

Table S1 Metabolome analysis of H-006-treated A549 cells.

## Data Availability

The data and materials used in the present study are available from the corresponding authors upon reasonable request.
