# Supplementary figures and images for: Identification of a dihydroorotate dehydrogenase inhibitor that inhibits cancer cell growth by proteomic profiling
Source: Oncol Res. 2023 Sep 15;31(6):833–44. doi: 10.32604/or.2023.030241 (PMC10513951; doi:10.32604/or.2023.030241)

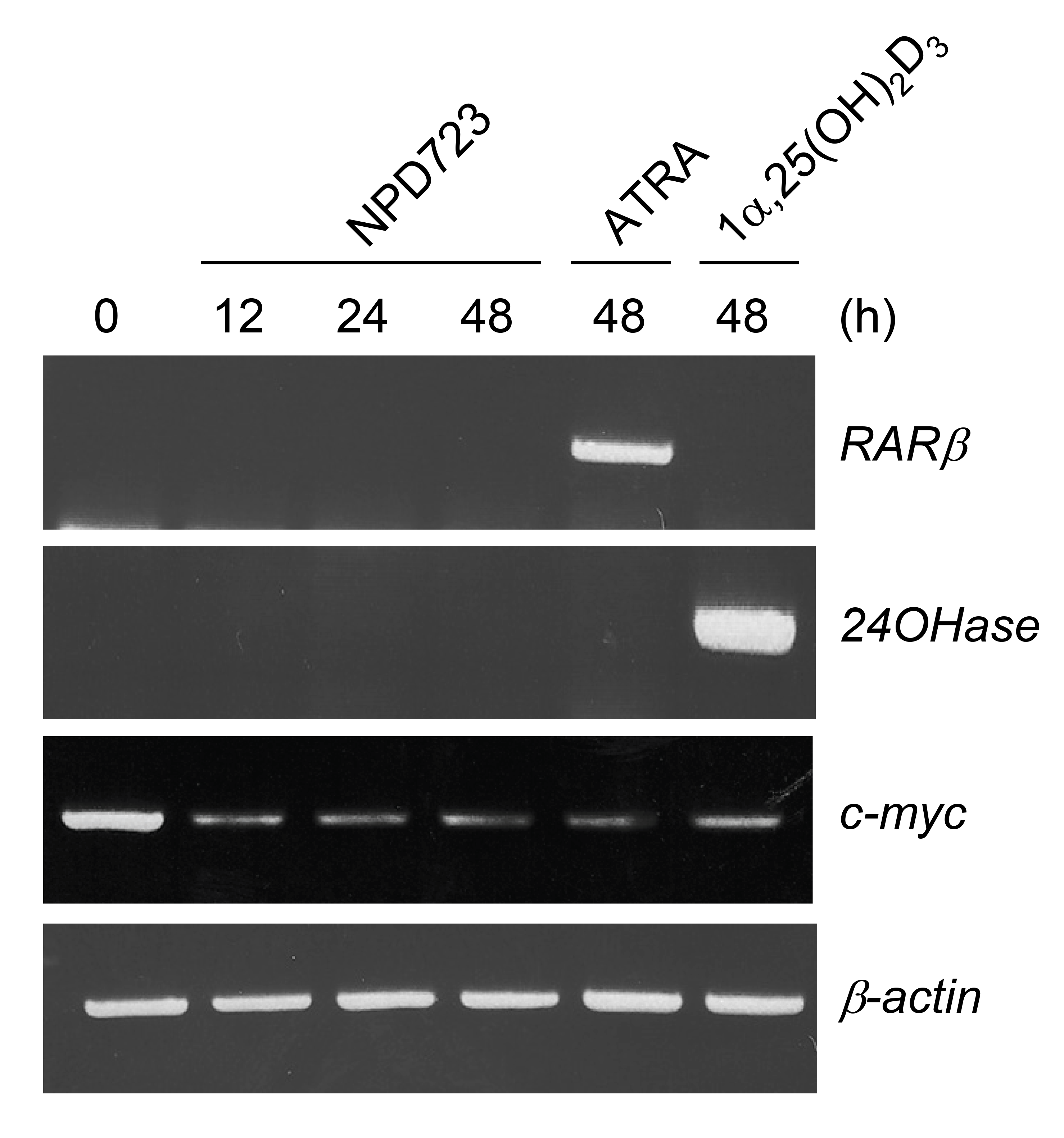

Supplement: FIGURE S1 [file OncolRes-31-30241-s001.tif]

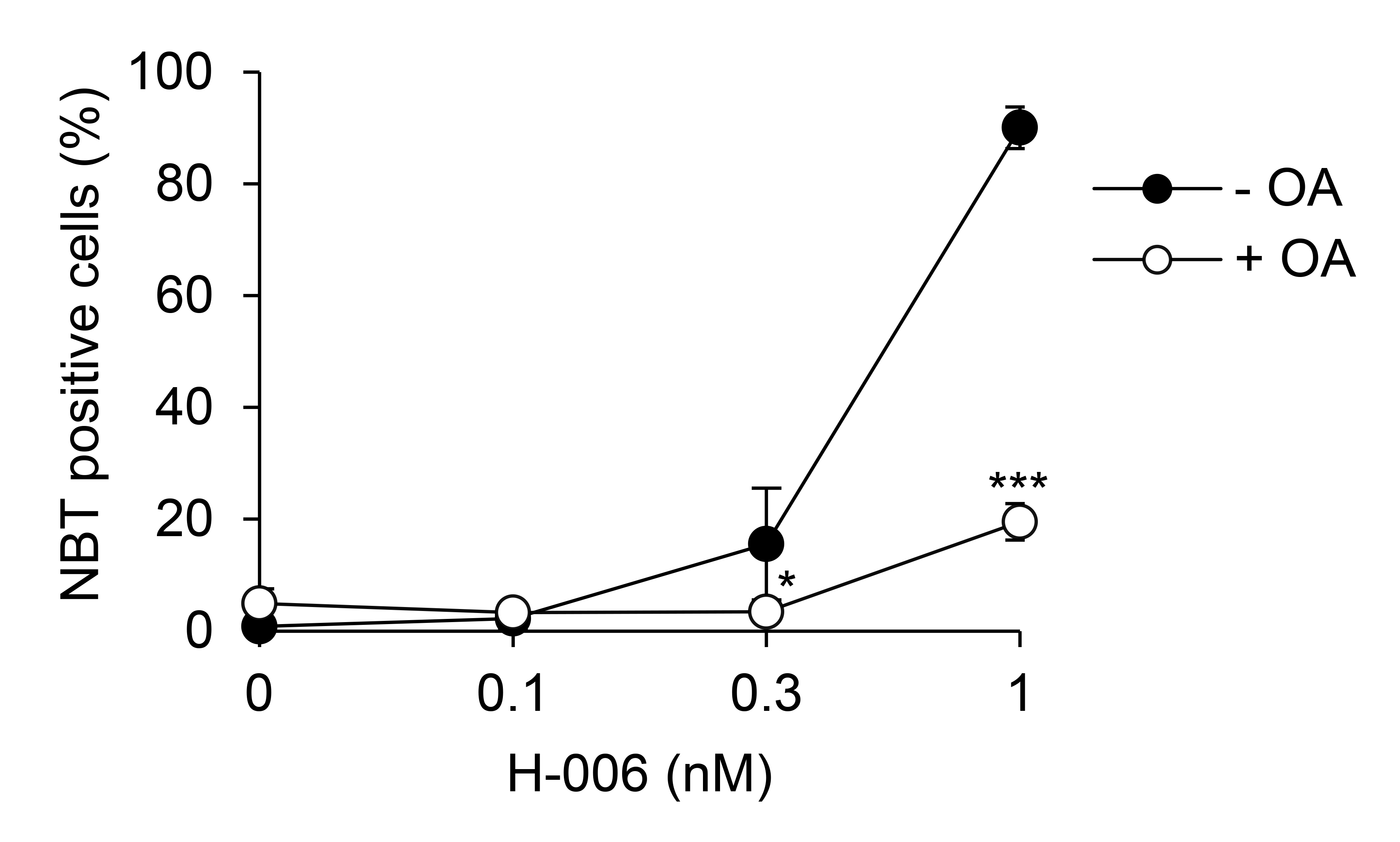

Supplement: FIGURE S5 [file OncolRes-31-30241-s005.tif]
